# New Delhi Metallo-Beta-Lactamase (NDM)-5 in Uropathogenic Klebsiella pneumoniae in a Tertiary Care Hospital in Jamaica

**DOI:** 10.1128/spectrum.03459-22

**Published:** 2023-01-31

**Authors:** Steven Stone, Camille-Ann Thoms-Rodriguez, Jenene Cameron, Christine Seah, Stacy Stephenson-Clarke, Roberto G. Melano

**Affiliations:** a The University of the West Indies, Mona, Jamaica; b The University Hospital of the West Indies, Mona, Jamaica; c Public Health Ontario Laboratory, Toronto, Ontario, Canada; d University of Toronto, Toronto, Ontario, Canada; University at Albany, State University of New York

**Keywords:** carbapenemase, UTI, antimicrobial resistance, Jamaica, NDM, urinary tract infection

## Abstract

We have investigated the prevalence of carbapenemase-producing uropathogens at the University Hospital of the West Indies, Jamaica. From 64 unique urine samples collected between January and March 2020, only 2 closely related Klebsiella pneumoniae (ST11, 14 SNPs of difference; no clear epidemiological links found between patients) were carbapenemase-producers. By whole-genome sequencing (WGS), *bla*_NDM-5_ was found on ~46 kb, IncX3 plasmid. These findings highlight the necessity for continuous surveillance of these pathogens in Jamaica.

**IMPORTANCE** As the problem of antibiotic resistance continues to be a global problem, we hope to be able to shed further insight into what is happening within the Caribbean, from which there has been a paucity of data. The ability to appropriately tackle the problem of resistance requires surveillance from all territories, including resource limited settings. In this paper, we look at a mechanism of resistance that renders some critical antibiotics useless, including carbapenems, cephalosporins, and penicillin.

## OBSERVATION

Given the limited surveillance data on carbapenemase-producing Enterobacterales in Jamaica, this study was conducted to investigate the prevalence of these enzymes in uropathogens, and compare findings to previous data in the country.

The study was conducted at the Bacteriology Laboratory in the Department of Microbiology, at the University Hospital of the West Indies in Jamaica. The study was cross-sectional, and conducted from January 2020 to March 2020. The study was based on a collection of 64 urinary isolates that met the inclusion criteria of the study (consecutive de-duplicated samples that contained a significant growth [>100, 000 CFU/mL] of Gram-negative bacteria; one type of isolate per patient considered during the period; isolates belonging to different species were included). Samples underwent identification by standard biochemical analyses. Kirby Bauer disc diffusion method was performed and interpreted, according to the Clinical and Laboratory Standards Institute guidelines (1). Suspected carbapenemase-producing isolates were tested by the modified carbapenem inactivation method (mCIM) to confirm carbapenemase activity (2). mCIM-positive isolates were first tested for the presence of carbapenemase genes by multiplex PCR (3, 4), and later by whole-genome sequencing (WGS), using the Illumina and Nanopore platforms, as previously described (5, 6). Approval was sought from, and granted by, the University of the West Indies Mona Campus Research Ethics Committee on January 8, 2020 (Code ECP 89, 19/20), inclusive of any necessary waivers of informed consent. The genome assemblies of the 2 isolates included in this study were deposited in GenBank and were registered under BioProject accession number PRJNA861980.

Samples were obtained from 80 patients that fell under the inclusion criteria for the study. However, due to unforeseen circumstances, only 64 isolates from 61 patients were successfully cultured for further testing, generating a 5.6% margin of error for the true population.

Table S1 shows a summary of the antibiograms. Only 2 isolates (3.12%) displayed carbapenemase activity, both were resistant to all the antimicrobial tested except gentamicin, and positive for *bla*_NDM_ gene by PCR. These carbapenemase-producing Klebsiella pneumoniae (GN4539 and GN4549) were recovered from one male and one female patients (76 and 65 years-old, respectively). They were treated by the internal medicine team, although they were never in the same ward. For the male patient, the infection was likely nosocomial (urosepsis), as he was admitted for >48h prior to sample collection. The female patient presented with sepsis. Her urine sample was collected at the accident and emergency department; however, she had been admitted to the medical floor just 9 days prior. Both patients were very ill, and were being treated for sepsis. The female patient died within 24 h of presentation, before the availability of culture results. She was administered ceftazidime. The male patient was administered ceftriaxone, but his medical records were incomplete, meaning that his clinical outcome was unknown. Given the spectrum of activity of NDM enzymes, it is evident that these treatments were sub-optimal.

Both samples of K. pneumoniae belonged to sequence type 11 (ST11). ST11 is part of the clonal complex 258 (CC258), together with ST258 and ST512, which have been disseminating *bla*_KPC_ genes successfully, particularly ST258. The majority of CC258 members are extremely resistant (XDR), due to an accumulation of a plethora of mechanisms, including plasmid-mediated (such as the ones described in our study), as well as chromosomal mutations, such as porin alterations (in *ompK35*, *ompK36*, and *ompK37*) or other genes causing colistin resistance (*mgrB*). The multinational distribution of ST11 disseminating *bla*_NDM_ genes would support the idea of this ST as an international epidemic carbapenem-resistant clone (7, 8). Core WGS analysis revealed a 14 SNPs variation between the 2 K. pneumoniae, which led us to suspect a common source of infection for both patients (Tables S2 and S3). However, they were not in the same ward, but they were treated with medicine in a medical ward. Staff from the medical team moving throughout all the medical wards, or a nosocomial environmental reservoir of this XDR clone, could be responsible of this dissemination, but the source was not identified in this study. Further investigation would be needed to avoid the potential for future outbreaks.

**FIG 1:**
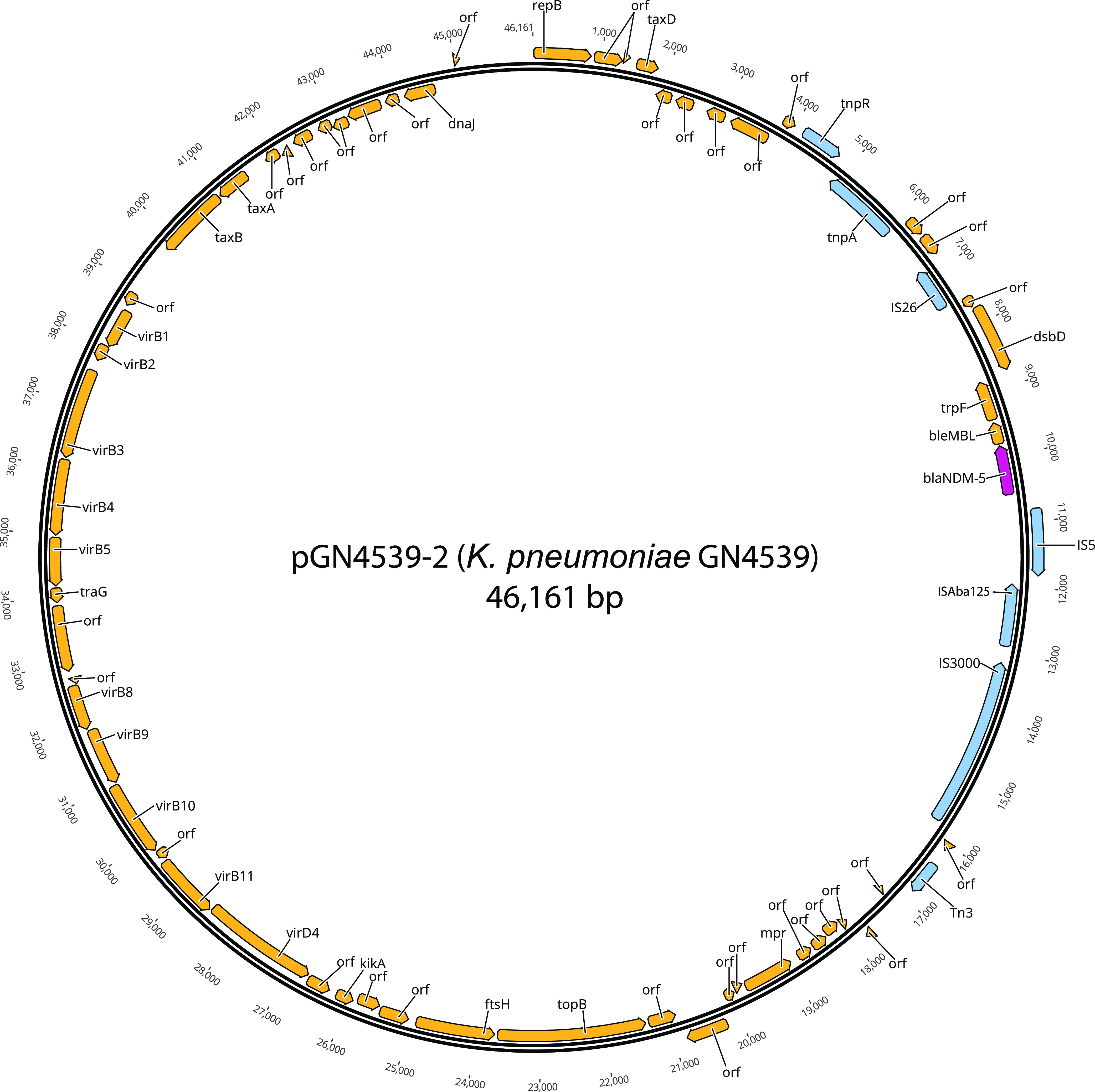
Plasmids harboring *bla*_NDM-5_ (pink) found within carbapenemase producing K. pneumoniae.

Both isolates had the expected chromosomal mechanisms of resistance to ß-lactams (*bla*_SHV-89_), fosfomycin (*fosA*), and low resistance to different drugs, including quinolones (*oqxAB*), as well as some missense mutations, such as in the QRDR regions of *gyrA* and *parC*, conferring high level resistance to fluoroquinolones (Table S4). Using the hybrid Illumina/Nanopore sequences, we could circularize plasmids, 3 of which harbored all the antimicrobial resistance genes found in these isolates (Fig. 1 and Fig. S1). An IncX3 plasmid of 46,161-bp was identified, harboring only *bla*_NDM-5_. This plasmid was identical or highly similar (99%) to others detected in E. coli (e.g., pR15_NDM-5, pJN05NDM-7, and pEC26-NDM-5 from China, GenBank accession numbers MH523639, MK256964, and CP060883, respectively; pEC08_NDM5 from Thailand, LC521848; pKW53T-NDM from Kuwait, KX214669). The largest IncFIB(K) plasmid (~217.5 kb) carried resistance to ß-lactams (*bla*_CTX-M-15_, *bla*_OXA-1_), quinolones (*qnrB1*, *aac(6′)-Ib-cr*), aminoglycosides (*aac(6′)-Ib-cr*), trimethoprim (*dfrA14*), tetracycline (*tetA*), and chloramphenicol (*catB3*). Another IncR plasmid of ~39 kb harbored resistance for sulfisoxazole (*sul1*), rifampicin (*AAR-3*), macrolides (*mphA*), quaternary ammonium compounds (*qacE*), as well as another copy of *bla*_OXA-1_, *aac(6′)-Ib-cr*, and *catB3*.

*bla*_NDM-5_ was the only carbapenemase gene found among the isolates screened. In 2012, an XDR K. pneumoniae ST336 carrying *bla*_NDM-1_ on a ~110 kb IncA/C plasmid was detected in Jamaica, but no additional surveillance data was generated since then (9, 10). Of concern is the limited formulary available for the conventional treatment of such XDR uropathogens, particularly in our setting. The NDM-5-producing isolates within our study population were only susceptible to gentamicin, often used in combination with a carbapenem as salvage therapy. Occasionally, polymyxin B is used empirically as susceptibility testing (using broth dilution is not conducted). Unfortunately, the aminoglycosides and the polymyxins are associated with significant toxicities. Newer agents, like cefiderocol, ceftazidime-avibactam, imipenem-cilastin-relebactam, and meropenem-vaborbactam, are not available locally, and often take too long to be imported on a case-by-case basis to be sufficiently impactful. Additionally, there is limited availability of tigecycline, the cost of which is prohibitive for most patients. Limitations of this study include a lack of more carbapenems for a more comprehensive antibiogram, as well as difficulties in retrieving complete clinical data, leading to an inability to extensively compare them.

This study highlights a low prevalence (~3%) of carbapenemase-producing uropathogens in our hospital. However, the finding of a clonal dissemination of the successful XDR ST11 raises a worrying alarm, further enhanced by a paucity of antimicrobial options for treatment in Jamaica. Studies like ours are fundamental to reveal the actual prevalence of antimicrobial resistance, which can guide the empirical treatment using local, updated information. Also, surveillance data can uncover the potential public health catastrophe if new antibiotics are not approved, as potential options for treatment of infections produced by pathogens as the ones described here. Having few alternative antibiotics for therapy, stacked against an increase in the prevalence of carbapenemases raises, creates an opportunity for poor outcomes. As such, further work should investigate the variants of the genes observed, as well as the virulence of the genes to aid in antibiotic therapy, and reduce the level of resistance in Jamaica.
