# Supplementary material for: New Delhi Metallo-Beta-Lactamase (NDM)-5 in Uropathogenic Klebsiella pneumoniae in a Tertiary Care Hospital in Jamaica
Source: Microbiol Spectr. 2023 Jan 31;11(2):e03459-22. doi: 10.1128/spectrum.03459-22 (PMC10100667; doi:10.1128/spectrum.03459-22)

NEW DELHI METALLO-BETA-LACTAMASE (NDM)-5 IN UROPATHOGENIC *Klebsiella pneumoniae* IN A TERTIARY CARE HOSPITAL IN JAMAICA

Steven Stone[1†], Camille-Ann Thoms-Rodriguez[1,2,†,*], Stacy Stephenson-Clarke[1], Jenene Cameron[2], Christine Seah[3], Roberto G. Melano[3,4],
†These authors share first authorship

[1]University of the West Indies, Mona, Jamaica
[2]University Hospital of the West Indies, Mona, Jamaica
[3]Public Health Ontario Laboratory, Canada
[4]University of Toronto, Canada

***Corresponding Author**:
Camille Ann Thoms Rodriguez
Department of Microbiology
The University of the West Indies
Mona, KGN 7; Jamaica; W.I.
camille.thomsrodriguez@uwimona.edu.jm
1-876-9772206

SUPPLEMENTARY FILES (FIGURES)

Supplementary Figure 1. Plasmids harboring antimicrobial resistance determinants (in pink) in one of the two *K. pneumoniae* (GN4539) harboring $bla_{\text{NDM-5}}$. The other (GN4549) harbored the same AMR plasmids.

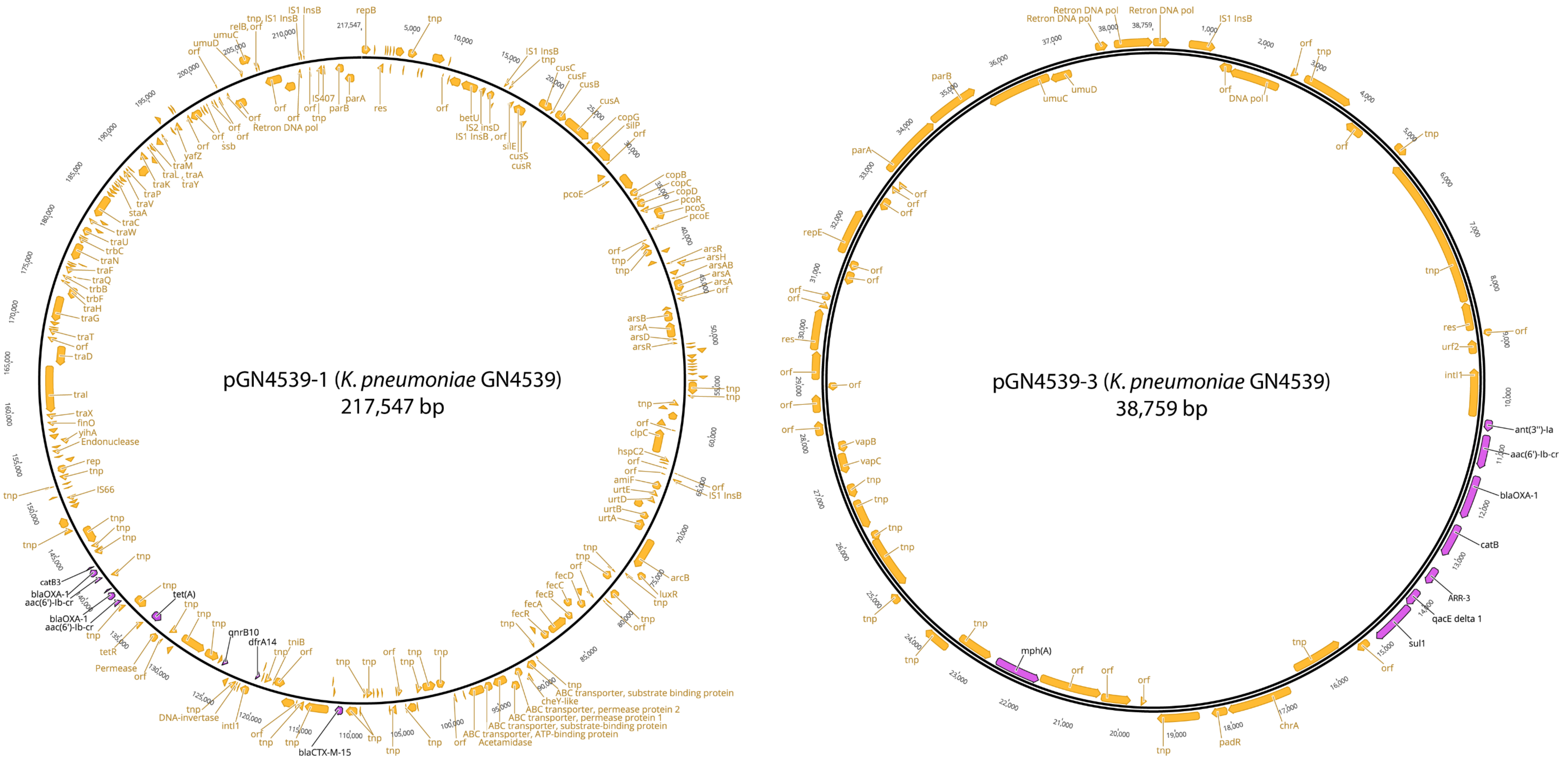

Supplement: Supplemental file 1 — Supplemental material. Download spectrum.03459-22-s0001.pdf, PDF file, 1.0 MB [file spectrum.03459-22-s0001.pdf]
